# Experimental Evidence for Adaptation to Species-Specific Gut Microbiota in House Mice

**DOI:** 10.1128/mSphere.00387-19

**Published:** 2019-07-10

**Authors:** Andrew H. Moeller, João C. Gomes-Neto, Sara Mantz, Hatem Kittana, Rafael R. Segura Munoz, Robert J. Schmaltz, Amanda E. Ramer-Tait, Michael W. Nachman

**Affiliations:** a Miller Institute for Basic Research in Science, University of California, Berkeley, California, USA; b Museum of Vertebrate Zoology, University of California, Berkeley, California, USA; c Department of Integrative Biology, University of California, Berkeley, California, USA; d Department of Food Science and Technology, University of Nebraska—Lincoln, Lincoln, Nebraska, USA; e Nebraska Food for Health Center, University of Nebraska—Lincoln, Lincoln, Nebraska, USA; University of Wisconsin—Madison

**Keywords:** evolutionary biology, metagenomics, microbial ecology

## Abstract

The gut microbial communities of mammals have codiversified with host species, and changes in the gut microbiota can have profound effects on host fitness. Therefore, the gut microbiota may drive adaptation in mammalian species, but this possibility is underexplored. Here, we show that the gut microbiota has codiversified with mice in the genus *Mus* over the past ∼6 million years, and we present experimental evidence that the gut microbiota has driven adaptive evolution of the house mouse, Mus musculus *domesticus*. Phylogenetic analyses of metagenome-assembled bacterial genomic sequences revealed that gut bacterial lineages have been retained within and diversified alongside *Mus* species over evolutionary time. Transplantation of gut microbiotas from various *Mus* species into germfree *M. m. domesticus* showed that foreign gut microbiotas slowed growth rate and upregulated macrophage inflammatory protein in hosts. These results suggest adaptation by *M. m. domesticus* to its gut microbiota since it diverged from other *Mus* species.

**IMPORTANCE** The communities of bacteria that reside within mammalian guts are deeply integrated with their hosts, but the impact of this gut microbiota on mammalian evolution remains poorly understood. Experimental transplantation of the gut microbiota between mouse species revealed that foreign gut microbiotas lowered the host growth rate and upregulated the expression of an immunomodulating cytokine. In addition, foreign gut microbiotas increased host liver sizes and attenuated sex-specific differences in host muscle and fat content. These results suggest that the house mouse has adapted to its species-specific gut microbiota.

## Abstract (precis)

The communities of bacteria that reside within mammalian guts are deeply integrated with their hosts, but the impact of this gut microbiota on mammalian evolution remains poorly understood. Experimental transplantation of the gut microbiota between mouse species revealed that foreign gut microbiotas lowered the host growth rate and upregulated the expression of an immunomodulating cytokine. In addition, foreign gut microbiotas increased host liver sizes and attenuated sex-specific differences in host muscle and fat content. These results suggest that the house mouse has adapted to its species-specific gut microbiota.

## INTRODUCTION

The gut bacterial communities of mammals can affect host health and fitness via interactions with metabolic (1), immune (2), and neuroendocrine (3) systems. Over evolutionary time, gut bacterial lineages and microbiota compositions have codiversified with mammalian species (1–18). For example, the evolutionary relationships among gut bacterial lineages recovered from humans and African apes correspond to the evolutionary relationships among their hosts (18), and compositional differences between the gut microbiotas of hominid species are positively associated with host divergence times (12). Given the myriad effects of the gut microbiota on hosts, changes in the gut microbiota may select for changes in host species that maximize fitness in the context of their new microbial environment. Under this scenario, adaptive divergence between mammalian species is shaped in part by changes in the gut microbiota.

This hypothesis generates predictions that can be tested experimentally. First, hosts are expected to display higher fitness when colonized with their indigenous gut microbiota than when colonized with the gut microbiota of a different host species. Second, reductions in host fitness caused by foreign gut microbiotas are expected to be proportional to the evolutionary time separating the host species. There is experimental evidence for adaptation to the gut microbiota in *Nasonia* wasps: germfree wasps inoculated with the gut microbiota of a host of a different species displayed reduced survival relative to hosts inoculated with their endogenous gut microbiota (19). Similarly, germfree mice inoculated with human microbiota display stunted development of T-cell diversity relative to germfree mice inoculated with mouse microbiota (20), suggesting that mice have adapted to their native gut microbiota. However, the possibility that closely related mammalian species have adapted to their species-specific gut microbiotas has not been tested by experiments with gnotobiotic hosts. Experiments with conventionally reared (i.e., not germfree) *Peromyscus* mice showed that hosts display reduced digestive performance when inoculated with the gut microbiotas of hosts of different species in some cases (19). This observation could indicate the adaptation of *Peromyscus* lineages to their species-specific gut microbiotas, but it could also reflect acclimation of *Peromyscus* individuals to the gut microbiota with which they developed.

## RESULTS AND DISCUSSION

**FIG 1:**
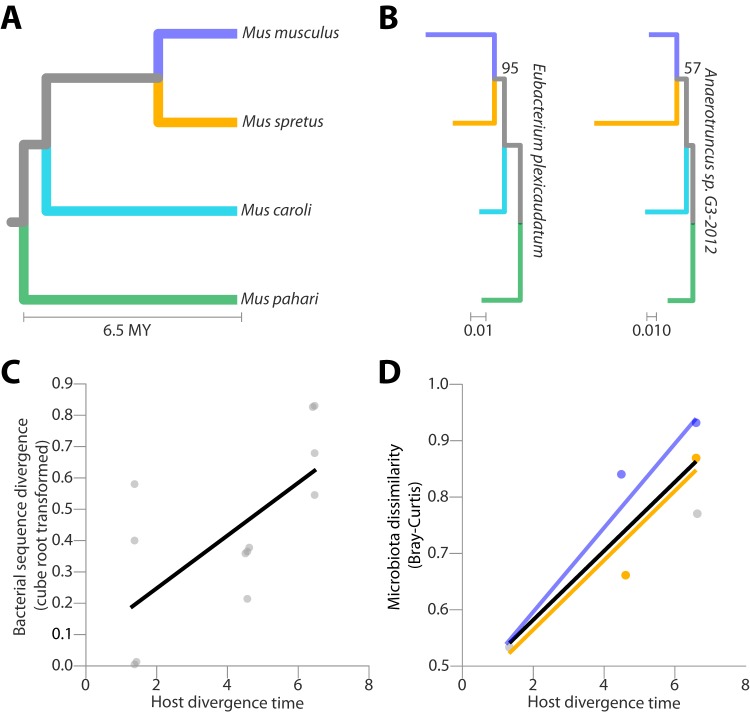
Codiversification of gut microbiota with *Mus* species. (A) Phylogeny of *Mus* species (drawn using TimeTree [21]). MY, millions of years. (B) Phylogenies of gut bacterial species for which homologous genomic regions were recovered from each *Mus* species. The bootstrap support value for each four-taxon topology is included next to each tree. For a four-taxon phylogeny, there are only three possible topologies, and bootstrap support can be calculated for only a single node, because defining one node defines the entire tree. Branches of bacterial phylogenies are colored to indicate the host species from which they were recovered corresponding to the coloring scheme used in panel A. (C) Scatter plot displays sequence divergence among closely related bacterial strains recovered from *Mus* species as a function of host divergence time. Each point represents the sequence divergence between two homologous strains of the same bacterial species recovered from different *Mus* species. Significance of non-zero slope of regression line, *P* value < 0.01. (D) Scatter plot displays genus-level microbiota dissimilarity (Bray-Curtis) as a function of host divergence time. Colored lines indicate best-fit regressions for pairwise comparisons containing Mus musculus *domesticus* microbiota (blue) or *Mus spretus* microbiota (yellow), both of which were compared to one another and to the *Mus pahari* microbiota. The black line represents best-fit regression for all pairwise comparisons. Significance of nonzero slope of black regression line, *P* value < 0.05.

We sought to develop an experimental system with which to test how the gut microbiota shapes the adaptive evolution of a mammalian host. We acquired wild-derived inbred mouse lines of Mus musculus *domesticus* (LEWES/EiJ), Mus spretus (SPRET/EiJ), Mus caroli (CAROLI/EiJ) and Mus pahari (Mus pahari/EiJ) from the Jackson Laboratory (see Text S1 in the supplemental material). A phylogeny of these *Mus* species retrieved from reference 21 is presented in Fig. 1A. In contrast to standard lines of laboratory mice, these wild-derived lines have never been rederived under sterile conditions. LEWES/EiJ, SPRET/EiJ, CAROLI/EiJ, and Mus pahari/EiJ were housed at the Jackson Laboratory for 26, 86, 26, and 32 generations, respectively. The mouse lines were moved to the University of California, Berkeley, and bred for one generation on identical diets (Teklad Global food, 18% Protein 6% Fat Rodent Diet) in the same room, and metagenomes from fecal samples collected from second-generation mice were sequenced on an Illumina NextSeq instrument (see Fig. S1 to S4 in the supplemental material). We were first interested in assessing whether bacterial lineages codiversified with their hosts. Metagenomic data were used to assemble contiguous sequences (contigs) from bacterial genomes. Homologous contigs for two bacterial species were detected in all four *Mus* species (Eubacterium plexicaudatum and *Anaerotruncus* sp. strain G3-2012). Phylogenetic analyses supported the hypothesis that these gut bacterial strains have codiversified with *Mus* species. The phylogenies of bacterial strains recovered from all four *Mus* species mirrored the host phylogeny (Fig. 1B). In addition, for all bacterial species for which homologous contigs were recovered from only three *Mus* species, sequence divergence between strains recovered from different *Mus* species was positively associated with the evolutionary time separating the hosts (significance of non-zero-slope *P* value = 0.0090; *R*^2^ = 0.344) (Fig. 1C). These bacterial species include Lactobacillus reuteri, Bacteroides uniformis, *Bacteroides* sp. strain 3-1-40A, *Bacteroides* sp. strain 2-1-22, and *Bacteroides* sp. strain 4-1-36. Therefore, wild-derived inbred lines of M. m. domesticus, M. spretus, M. caroli, and M. pahari harbor host species-specific gut bacterial lineages that reflect the evolutionary relationships of their hosts and that were stably maintained in mouse facilities for dozens of generations. These results extend previous observations that components of wild-derived gut microbiotas can be maintained within conventionally reared inbred mouse lines for >10 generations (22), despite the effects of captivity on components of the microbiota (23, 24). We also observed that the genus-level compositional profiles of gut microbiotas of the four *Mus* species recapitulated the host phylogeny. Bray-Curtis dissimilarities among the genus-level compositional profiles of the gut microbiotas of the four *Mus* species were positively associated with the evolutionary time separating the hosts (Fig. 1D, significance of non-zero-slope *P* value = 0.0305; *R*^2^ = 0.729). These results mirror previous observations of concordance between host phylogenies and dendrograms of microbiota composition (6, 19). Together, our results indicate that some host species-specific members of the wild gut microbiota of *Mus* species have been maintained in captivity.

10.1128/mSphere.00387-19.1 FIG S1 Taxon abundance plot for the *M. m. domesticus* donor sample generated by MG-RAST (40). Download FIG S1, PDF file, 0.2 MB. Copyright © 2019 Moeller et al. 2019 Moeller et al. This content is distributed under the terms of the Creative Commons Attribution 4.0 International license.

10.1128/mSphere.00387-19.2 FIG S2 Taxon abundance plot for the *M. spretus* donor sample generated by MG-RAST (40). Download FIG S2, PDF file, 0.2 MB. Copyright © 2019 Moeller et al. 2019 Moeller et al. This content is distributed under the terms of the Creative Commons Attribution 4.0 International license.

10.1128/mSphere.00387-19.3 FIG S3 Taxon abundance plot for the *M. caroli* donor sample generated by MG-RAST (40). Download FIG S3, PDF file, 0.2 MB. Copyright © 2019 Moeller et al. 2019 Moeller et al. This content is distributed under the terms of the Creative Commons Attribution 4.0 International license.

10.1128/mSphere.00387-19.4 FIG S4 Taxon abundance plot for the *M. pahari* donor sample generated by MG-RAST (40). Download FIG S4, PDF file, 0.2 MB. Copyright © 2019 Moeller et al. 2019 Moeller et al. This content is distributed under the terms of the Creative Commons Attribution 4.0 International license.

10.1128/mSphere.00387-19.10 TEXT S1 Supplemental methods and results. Download Text S1, PDF file, 0.1 MB. Copyright © 2019 Moeller et al. 2019 Moeller et al. This content is distributed under the terms of the Creative Commons Attribution 4.0 International license.

**FIG 2:**
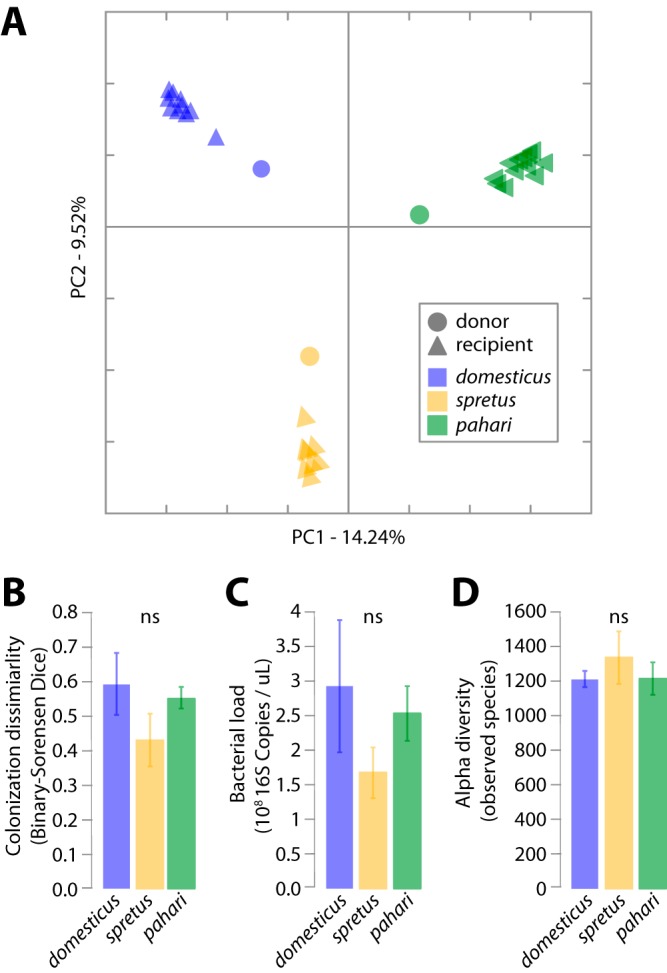
Successful transplantation of gut microbiotas of *Mus* species into Mus musculus *domesticus*. The three *Mus* species shown are *M. m. domesticus, M. spretus*, and *M. pahari.* (A) Principal coordinate plot of Bray-Curtis dissimilarities among the gut microbiotas of microbiota donors (circles) and recipients (triangles). Circles and triangles are colored based on the host species of origin corresponding to the colors shown in Fig. 1A. (B to D) Bar plots display colonization success as measured by binary Sorensen-Dice dissimilarity between donor and recipient (B), bacterial load in recipients (C), and alpha diversity in recipients (D). Bars are colored based on host species of origin corresponding to the colors shown in Fig. 1A. Significance of nonparametric permutation tests and *t* tests are shown as not significant (ns) (*P* value > 0.05).

To test whether *M. m. domesticus* is adapted to its species-specific gut microbiota, we conducted microbiota transplant experiments in germfree mice. We transplanted the microbiotas of *M. m. domesticus*, *M. spretus*, and *M. pahari* into three groups of germfree *M. m. domesticus* (C57BL/6) (*n* = 10, 8, and 13, respectively) when the mice were 10 days old. In contrast to microbiota transplantation experiments into mice that developed in the presence of an uncontrolled microbiota, microbiota transplant experiments in germfree hosts allow explicit tests for adaptation of host lineages to the microbiota (25). We first tested whether the microbiotas of each *Mus* species successfully colonized *M. m. domesticus*. Previous experiments observed that gut bacteria from chickens displayed lower fitness in the mouse gut than did gut bacteria from mice (26). In contrast, we found that the transplantation of total microbiotas from the three wild-derived *Mus* species into germfree *M. m. domesticus* was comparably successful in every treatment (Fig. 2). 16S ribosomal DNA (rDNA) was sequenced from fecal samples from all donor and recipient mice (Text S1 and Table S1). Host species-specific microbiota compositions were maintained in the recipients into adulthood (Fig. 2A and Text S1). Adult recipient *M. m. domesticus* harbored gut microbiotas that were more compositionally similar to the microbiota of their microbiota donor than to the microbiota of any other donor (Fig. 2A; nonparametric permutation test *P* value < 0.001 for each treatment). In addition, the community compositions of recipient *M. m. domesticus* reflected the evolutionary relationships among the donors (Fig. S5 and Text S1). For example, gnotobiotic mice receiving the *M. m. domesticus* microbiota displayed microbiota compositions that were more similar to the microbiota of recipients of the *M. spretus* microbiota than the microbiota of recipients of the *M. pahari* microbiota. Microbiota colonization success, as measured by binary Sorensen-Dice microbiota dissimilarity between donor and recipient mice (Text S1), was not significantly different among treatments (Fig. 2B). Moreover, measuring colonization success for each bacterial genus individually revealed no bacterial genera that colonized significantly better in the *M. m. domesticus* treatment than in the *M. spretus* and *M. pahari* treatments (Text S1). In addition, the total bacterial load in fecal samples, as measured by quantitative PCR of 16S rRNA gene copy number, was comparable across treatments (Fig. 2C). Similarly, individual recipient mice harbored roughly equivalent numbers of bacterial lineages regardless of treatment (Fig. 2D). We also found that phylum-level compositional microbiome profiles of the three experimental groups of recipient *M. m. domesticus* were not more compositionally similar to one another than were the profiles of the donor species (Fig. S6 and Text S1), contrasting results of previous gnotobiotic microbiome transplants between zebrafish and mice (27). These findings demonstrate that compositional differences between the gut microbiotas of closely related *Mus* species can be maintained in a common host genetic background and indicate the successful colonization of each *Mus* microbiota in germfree *M. m. domesticus*.

10.1128/mSphere.00387-19.5 FIG S5 (A) Phylogeny of *Mus* species. The scale bar indicates divergence time in millions of years (MY). (B) Dendrogram of binary Sorensen-Dice dissimilarities among donors and recipients. The scale bar indicates binary Sorensen-Dice dissimilarity. In panels A and B, branches are colored corresponding to the colors shown in Fig. 1. Download FIG S5, PDF file, 0.06 MB. Copyright © 2019 Moeller et al. 2019 Moeller et al. This content is distributed under the terms of the Creative Commons Attribution 4.0 International license.

10.1128/mSphere.00387-19.6 FIG S6 Scatterplot displays the phylum-level Bray-Curtis dissimilarities between the microbiotas of recipient mice (left) and between the microbiotas of donor mice (right). Mean of each group is denoted by a horizontal line. Significance of difference between means is indicated as “ns” (for not significant) by *t* test of logit transformed data (*P* value > 0.05). Download FIG S6, PDF file, 0.04 MB. Copyright © 2019 Moeller et al. 2019 Moeller et al. This content is distributed under the terms of the Creative Commons Attribution 4.0 International license.

10.1128/mSphere.00387-19.7 TABLE S1 Metadata for fecal samples. Download Table S1, XLSX file, 0.01 MB. Copyright © 2019 Moeller et al. 2019 Moeller et al. This content is distributed under the terms of the Creative Commons Attribution 4.0 International license.

**FIG 3:**
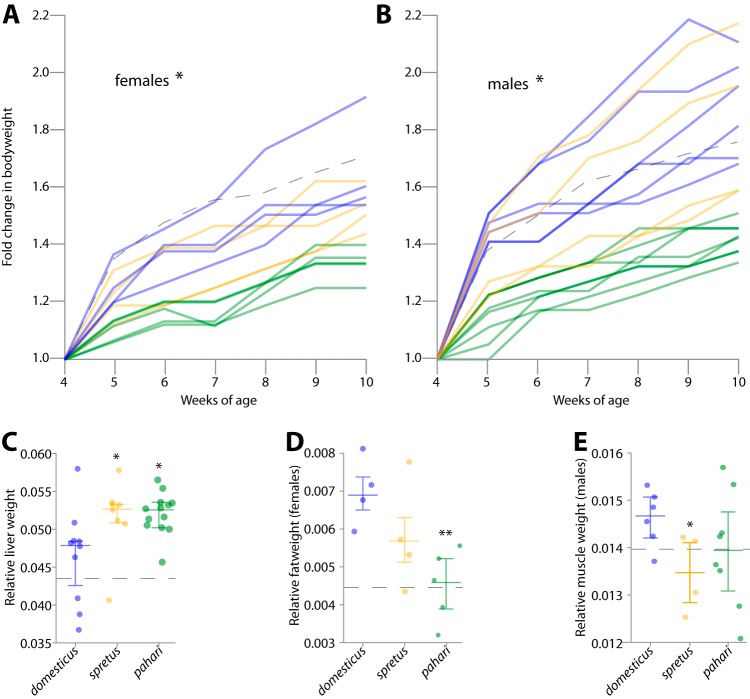
Foreign microbiotas slow host growth rate and disrupt organismal phenotypes. (A and B) Line graphs display growth rates of recipient *M. m. domesticus* from 4 to 10 weeks of age for males (*n* = 18) (A) and females (*n* = 13) (B). The lines are colored by donor host species corresponding to the colors in Fig. 1A. Dashed lines indicate mean body weight of germfree mice (*n* = 16). The significance of microbiota treatment on fold change in body weight at 10-week-old mice is shown (*, likelihood ratio test *P* value < 0.05 [Text S1]). (C to E) Dot plots display relative weight of livers in male and female recipient mice (C), gonadal adipose tissues in female mice (D), and gastrocnemius, soleus, and tibialis muscles in male mice (E).The three *Mus* species shown are *M. m. domesticus, M. spretus*, and *M. pahari.* The colors indicate donor host species corresponding to the colors shown in Fig. 1A. The solid horizontal lines indicate the means and first and third quartiles, and dashed horizontal lines indicate mean trait values of germfree mice. Values that are significantly different by *t* test are indicated by asterisks as follows: *, *P* value < 0.05; **, *P* value < 0.01.

We observed several lines of evidence suggesting that *M. m. domesticus* has evolved in response to its microbiota since it diverged from other *Mus* species. We measured mouse body size over time in each treatment in order to test whether host growth rate was negatively associated with the evolutionary distance between donor and recipient hosts. Postweaning body weight increases are associated with larger litter sizes in lab mice (28), and body size affects success in dominance hierarchies and metabolic investment in offspring, both of which are key components of mouse fitness in the wild (29). *M. m. domesticus* inoculated with an *M. spretus* or *M. pahari* microbiota displayed a lower mean growth rate than did *M. m. domesticus* inoculated with an *M. m. domesticus* microbiota (Fig. 3A and B and Table S2). On the basis of comparisons between treatments of mean fold change in body weight for 10-week-old mice, the recipients of the *M. m. domesticus* microbiota outgrew recipients of the *M. spretus* microbiota as well as recipients of the *M. pahari* microbiota, on average. This pattern was observed in both males (Fig. 3A) and females (Fig. 3B). Comparisons of linear mixed-effects models controlling for sex, cage, and initial body weight indicated a significant effect of microbiota treatment on the fold change in body weight at 10 weeks in both males and females (likelihood ratio test *P* value < 0.05; Text S1). In addition, these tests supported the hypothesis that the *M. pahari* microbiota had a larger effect on the growth of gnotobiotic *M. m. domesticus* than did the *M. spretus* microbiota (Text S1). Of the 36 possible ways to order mean fold change in body weight in the males and females of the three treatments, the observed pattern, with *M. m. domesticus* above *M. spretus* above *M. pahari* in both sexes, is the single configuration predicted if *M. m. domesticus* has adapted to its gut microbiota since diverging from other *Mus* species. Moreover, recipients of the *M. pahari* gut microbiotas displayed reduced mean fold change in body weight at 10 weeks relative to germfree mice (*t* test *P* value < 0.01), suggesting deleterious effects of *M. pahari* gut microbiotas on *M. m. domesticus* relative to the absence of a gut microbiota. These differences in host growth rate could not be attributed to differences in food consumption, which did not differ significantly between treatments (Text S1).

10.1128/mSphere.00387-19.8 TABLE S2 Body, tissue, and organ weights of recipient mice. Download Table S2, XLSX file, 0.01 MB. Copyright © 2019 Moeller et al. 2019 Moeller et al. This content is distributed under the terms of the Creative Commons Attribution 4.0 International license.

It remains unclear whether the differential growth rate effects of native and foreign microbiotas are due to differences in community composition between foreign and native gut microbiotas (e.g., differences in the relative abundances of phyla) or to genetic differences between related microbial strains present in foreign and native gut microbiotas (e.g., those that have codiversified with host species). Comparing the relative abundances of bacterial genera across treatments (Table S3, sheet 1) revealed no bacterial genera whose relative abundances were both significantly different between treatments and associated with host growth rate. This result suggests that fine-scale genetic differences among gut bacteria recovered from *Mus* species may underlie the differential effects of foreign and indigenous gut microbiotas on the growth rate of *M. m. domesticus*.

10.1128/mSphere.00387-19.9 TABLE S3 Relative abundances of bacterial genera in donor and recipient mice (sheet 1). Cytokine and chemokine abundances in sea of recipient mice (sheet 2). Download Table S3, XLSX file, 0.04 MB. Copyright © 2019 Moeller et al. 2019 Moeller et al. This content is distributed under the terms of the Creative Commons Attribution 4.0 International license.

To further investigate how host growth was affected by indigenous and foreign gut microbiotas, we measured tissue and organ sizes of all gnotobiotic mice in each treatment. *M. m. domesticus* colonized with a foreign microbiota displayed enlarged livers at 10 weeks of age relative to *M. m. domesticus* colonized with its indigenous microbiota (Fig. 3C) and relative to germfree mice (*t* test *P* value < 0.05 in each comparison). Our experiments were unable to determine the consequences of liver enlargement on the health or fitness of gnotobiotic mice, but in humans, an enlarged liver, i.e., hepatomegaly, has been associated with infection (30), metabolic syndrome (31), or autoimmune disease (32). In addition to enlarged livers, we also observed several sex-specific effects. *M. spretus* and *M. pahari* microbiotas were associated with reduced muscle content in males and fat content in females relative to the *M. m. domesticus* microbiota (Fig. 3D and E). In contrast, we did not observe reductions in muscle content in females or in fat content in males in these treatments (Table S2). In addition, female and male mice inoculated with the *M. m. domesticus* microbiota displayed increased fat and muscle weight, respectively, relative to germfree mice (*t* test *P* value < 0.05 in each comparison), but these significant effects were not observed in mice inoculated with foreign microbiotas. These observations indicate a role for the *M. m. domesticus*-specific gut microbiota in the postnatal development of body composition in male and female *M. m. domesticus*.

**FIG 4:**
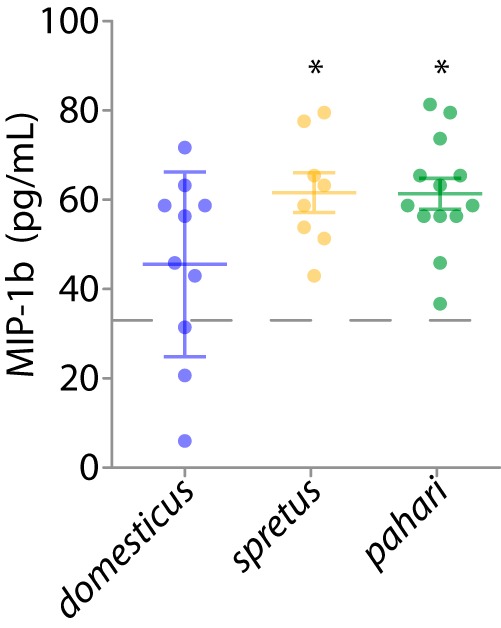
Foreign microbiotas upregulate production of macrophage inflammatory protein. Dot plots display concentrations in blood serum of macrophage inflammatory protein 1β (MIP-1β) (CCL3) in recipient mice at 10 weeks of age (*n* = 31). The three *Mus* species shown are *M. m. domesticus, M. spretus*, and *M. pahari.* Colors indicate donor host species corresponding to the colors shown in Fig. 1A. Horizontal lines indicate means and first and third quartiles. The dashed horizontal line indicates the mean *MIP1b* concentration in germfree negative-control mice (*n* = 9). Asterisks indicate significance of *t* tests against the germfree treatment (*, *P* value < 0.05). MIP-1β expression in the gnotobiotic mice inoculated with *M. m. domesticus* microbiota did not differ significantly from the germfree treatment (*t* test *P* value > 0.05).

Next, we tested whether gnotobiotic *M. m. domesticus* displayed differential immune responses when inoculated with either native or foreign gut microbiotas. We reasoned that upregulation of immune response by foreign gut microbiotas relative to the indigenous gut microbiota would be consistent with adaptation of *M. m. domesticus* to its indigenous gut microbiota. To examine this possibility, we assayed cytokine and chemokine expression in all gnotobiotic mice in each treatment. Quantifying abundances of 25 cytokines/chemokines in sera of recipient mice in each of the transplant treatments at 10 weeks of age (Table S3, sheet 2) indicated that *M. m. domesticus* colonized with an *M. spretus* or *M. pahari* microbiota displayed significantly altered immune response. Fisher’s combined probability test across all cytokines and chemokines indicated significant differences between the *M. m. domesticus* treatments and the *M. spretus* and *M. pahari* treatments (*P* value < 0.05 in both comparisons). False-discovery correction revealed a single chemokine, macrophage inflammatory protein 1β (MIP-1β/CCL3), that was differentially expressed in individuals colonized with the *M. m. domesticus* microbiota relative to individuals colonized with *M. spretus* or *M. pahari* microbiotas (Fig. 4). MIP-1β is released by macrophages in response to bacterial lipopolysaccharide (33), inducing the chemotaxis and adhesion of T cells (in particular CD8^+^ T cells) (34). In addition, experiments with colonic epithelial cell lines have shown that expression of this chemokine is induced by Escherichia coli, but not by commensal Bacteroides ovatus and Lactobacillus rhamnosus (35). The upregulation by foreign gut microbiotas of a chemokine associated with immune response is consistent with deleterious effects of foreign gut microbiotas on *M. m. domesticus*. However, these observations do not necessarily indicate a causal relationship between immune system activation by foreign gut microbiotas and the observed reductions in host growth rate (i.e., Fig. 3). Previous experiments have suggested that the immune system of *M. m domesticus* has adapted to its gut microbiota since diverging from humans ∼90 million years ago (20). Our results lend support to the possibility that the immune system of *M. m. domesticus* has adapted to its indigenous gut microbiota since the divergence of *M. m. domesticus* from other *Mus* species over the past ∼6 million years.

Our results support the hypothesis that *M. m. domesticus* has adapted to its gut microbiota. An alternative explanation is that the *M. m. domesticus* gut microbiota increases host growth rate relative to the *M. spretus* or *M. pahari* gut microbiota regardless of host genetic background and that the reductions in growth rate that we observe are not a consequence of an evolutionary mismatch between host and gut microbiota. Differentiating between these alternative hypotheses will require reciprocal transplant experiments between germfree *Mus* lineages. However, this alternative cannot explain the relationship between evolutionary distance between donor and recipient and growth rate reduction, nor can it explain the observation that *M. m. domesticus* harboring an *M. spretus* or *M. pahari* gut microbiota grew more slowly than germfree *M. m. domesticus*. In addition, the composition of the gut microbiota can vary widely within individual mice (36), between mouse populations (37), and between mouse subspecies (38). Therefore, our observations indicate a need for microbiota transplant studies between additional strains of the *Mus* species examined in the present study to further test the hypothesis that *M. m. domesticus* has adapted to its gut microbiota.

We found that wild-derived inbred laboratory lines of *Mus* species harbor compositionally distinct gut microbiotas that reflect the unique evolutionary histories of their hosts. These microbiotas include bacterial lineages that appear to have codiversified with their hosts. The postnatal development of *M. m. domesticus* hosts was disrupted by the foreign gut microbiotas of *M. spretus* and *M. pahari*. In addition, foreign gut microbiotas of *M. spretus* and *M. pahari* induced upregulated immune responses in *M. m. domesticus* hosts compared to hosts colonized by the indigenous *M. m. domesticus* gut microbiota. These results are consistent with the hypothesis that *M. m. domesticus* has adapted to its host-species specific gut microbiota since diverging from other *Mus* species.

## MATERIALS AND METHODS

### Animal husbandry and experimental protocol.

Two breeding pairs of LEWES/EiJ, SPRET/EiJ, CAROLI/EiJ, and Mus pahari/EiJ inbred mouse lines were obtained from the Jackson Laboratory. Lines were propagated for one generation in a shared room at the University of California, Berkeley, on sterilized Teklad Global food (18% Protein 6% Fat Rodent Diet) in accordance with an Animal Care and Use Committee (ACUC) protocol (AUP 2016-03-8548). Fecal samples from donor mice were collected at 20 weeks of age, flash frozen, stored at −80°C, and shipped on dry ice to the University of Nebraska—Lincoln. Fecal pellets were homogenized in sterile, prereduced phosphate-buffered saline (PBS) under anaerobic conditions, supplemented with 10% glycerol, and then stored at −80°C until administered to recipient mice.

Germfree C57BL/6 mice were born and reared in flexible film isolators and maintained under gnotobiotic conditions at the University of Nebraska—Lincoln. Breeding mice and pups (up to 21 days of age) were fed autoclaved LabDiet 5021 (Purina Foods, St. Louis, MO) *ad libitum*. After weaning (at 21 days of age), mice were fed autoclaved LabDiet 5K67 (Purina Foods) *ad libitum*. The germfree status of the breeding colony was confirmed routinely as described previously using culture, microscopy, and PCR (39). The Institutional Animal Care and Use Committee of the University of Nebraska—Lincoln approved all procedures involving animals (Project ID 1215). Microbiota transplant experiments included 8, 10, and 13 recipient mice for the *M. spretus*, *M. m. domesticus*, and *M. pahari* treatments, respectively.

A fecal slurry from an individual of each *Mus* species was introduced into one of three gnotobiotic isolators containing germfree C57BL/6 mouse breeding pairs nursing pups. One group of germfree mice was inoculated with PBS as a negative control (*n* = 16). Mice were inoculated orally at 10 ± 2 days after birth by introducing 50 μl of a fecal slurry into the mouth of each pup, with the leftover inoculum deposited on the parents’ fur and the cage bedding. Pups were weaned at 21 ± 1 days after birth, and the parents were euthanized. The pups were maintained in gnotobiotic isolators (18 males and 13 females) and placed into separate cages according to sex. The pups were weighed weekly, and their fecal pellets were collected. At 10 weeks of age, the recipient mice were euthanized, and their blood and tissues were harvested. Growth rate was calculated against the baseline body weights measured in 4-week-old mice.

### 16S rDNA sequencing and qPCR.

Genomic DNA was extracted from fecal pellets from all donor and 6-week-old recipient mice with a bead-beating procedure (MO BIO PowerFecal DNA kit). The V4-V5 region of bacterial 16S rDNA from each extraction was amplified, and amplicons were sequenced on an Illumina MiSeq as described previously to a depth of >10,000 reads per sample (12). Sample sizes and metadata are presented in Table S1 in the supplemental material. All 16S DNA extraction, library prep, and sequencing procedures were conducted at the Microbial Analysis, Resources, and Services (MARS) facility at the University of Connecticut. In addition to 16S sequencing, total genomic DNA from donor mice was sequenced at the Integrative Microbiome Resource at Dalhousie University. Quantitative PCR (qPCR) was performed on fecal samples collected from 6-week-old gnotobiotic mice (Table S1) on The BioMark Systems paired with the Fluidigm Dynamic Arrays at the University of North Carolina, Chapel Hill.

### Metagenomic sequencing and data processing.

Total genomic DNA from donor fecal pellets was extracted via a bead-beating procedure (MO BIO PowerFecal DNA kit), libraries were prepared using the Illumina Nextera Flex kit, and libraries were sequenced on an Illumina NextSeq at a depth of ∼3.5 Mb per sample, yielding ∼2.9 million reads, ∼4.1 million reads, ∼1.9 million reads, and ∼3.5 million reads for the *Mus pahari* donor, the *Mus caroli* donor, the *Mus spretus* donor, and the Mus musculus *domesticus* donor, respectively. All DNA extractions, library preps, and sequencing were performed at the Integrated Microbiome Resources at Dalhousie University. Reads from shotgun metagenomes were classified to the genus level in Mg-RAST v4.0.2 (40) using default parameters. Reads were mapped to bacterial species, and strain-level phylogenetic analyses were conducted using StrainPhlAn v1 (41) using default parameters. Phylogenetic analyses of bacterial strains were bootstrapped in MEGA 7.0 (42). Taxon abundance plots for *M. m. domesticus*, *M. spretus*, *M. caroli*, and *M*. *pahari* derived from metagenomic data are presented in Fig. S1, S2, S3, and S4, respectively.

### 16S data processing.

Sequences were filtered for quality and collapsed into 97% nucleotide similarity operational taxonomic units (OTUs) in QIIME 1.9 (43) using default parameters. OTUs represented by fewer than two reads or detected in fewer than two samples were discarded to eliminate sequencing errors. The remaining OTUs were assigned to taxonomic ranks against the Silva database using uclust, and unclassifiable OTUs were removed. For all downstream analyses, each sample was rarefied to a depth of 10,000 reads.

### Cytokine and chemokine quantification.

Chemokine and cytokine levels were measured using a Mouse Cytokine/Chemokine Magnetic Bead kit (Milliplex; Millipore, Billerica, MA) and a MAGPIX instrument (Luminex Corporation, Austin, TX) per the manufacturer’s instructions.

### Statistical analyses.

Bray-Curtis and binary Sorensen-Dice dissimilarities and principal coordinates for 16S rDNA data were calculated in QIIME (43). Binary Sorensen-Dice dissimilarities were employed to calculate colonization success of microbiotas in gnotobiotic mice, because this dissimilarity measure summarizes overlap between microbiotas in terms of presence and absence of gut bacterial lineages. All linear models were fit using the “stats” package in R v3.5.0. Nonparametric permutation tests were employed to test for significant differences among treatments in microbiome dissimilarity. In addition, *t* tests were employed to test for differences among treatments in bacterial load, alpha diversity, body size, tissue and organ weights, and cytokine/chemokine abundances. Bonferroni-corrected *P* values for cytokine comparisons were calculated. For cytokine/chemokine analyses, males and females were analyzed separately, and *P* values were combined using Fisher’s method for combined *P* values as implemented in the “metap” package in R v3.5.0.

### Data availability.

Metagenomic data are available in MG-RAST under accession no. 4783021.3, 4783019.3, 4783020.3, and 4783022.3. 16S rDNA data are available in the European Nucleotide Archive under accession no. PRJEB33256.

## References

[1] Turnbaugh PJ, Ley RE, Mahowald MA, Magrini V, Mardis ER, Gordon JI 2006 An obesity-associated gut microbiome with increased capacity for energy harvest. Nature 444:1027–1031. doi:10.1038/nature05414. 17183312

[2] Kau AL, Ahern PP, Griffin NW, Goodman AL, Gordon JI 2011 Human nutrition, the gut microbiome and the immune system. Nature 474:327–336. doi:10.1038/nature10213. 21677749 PMC3298082

[3] Cryan JF, Dinan TG 2012 Mind-altering microorganisms: the impact of the gut microbiota on brain and behaviour. Nat Rev Neurosci 13:701–712. doi:10.1038/nrn3346. 22968153

[4] Ley RE, Hamady M, Lozupone C, Turnbaugh PJ, Ramey RR, Bircher JS, Schlegel ML, Tucker TA, Schrenzel MD, Knight R, Gordon JI 2008 Evolution of mammals and their gut microbes. Science 320:1647–1651. doi:10.1126/science.1155725. 18497261 PMC2649005

[5] Muegge BD, Kuczynski J, Knights D, Clemente JC, González A, Fontana L, Henrissat B, Knight R, Gordon JI 2011 Diet drives convergence in gut microbiome functions across mammalian phylogeny and within humans. Science 332:970–974. doi:10.1126/science.1198719. 21596990 PMC3303602

[6] Ochman H, Worobey M, Kuo CH, Ndjango JB, Peeters M, Hahn BH, Hugenholtz P 2010 Evolutionary relationships of wild hominids recapitulated by gut microbial communities. PLoS Biol 8:e1000546. doi:10.1371/journal.pbio.1000546. 21103409 PMC2982803

[7] Yildirim S, Yeoman CJ, Sipos M, Torralba M, Wilson BA, Goldberg TL, Stumpf RM, Leigh SR, White BA, Nelson KE 2010 Characterization of the fecal microbiome from non-human wild primates reveals species specific microbial communities. PLoS One 5:e13963. doi:10.1371/journal.pone.0013963. 21103066 PMC2980488

[8] Amato KR, Yeoman CJ, Kent A, Righini N, Carbonero F, Estrada A, Gaskins HR, Stumpf RM, Yildirim S, Torralba M, Gillis M, Wilson BA, Nelson KE, White BA, Leigh SR 2013 Habitat degradation impacts black howler monkey (Alouatta pigra) gastrointestinal microbiomes. ISME J 7:1344–1353. doi:10.1038/ismej.2013.16. 23486247 PMC3695285

[9] Nelson TM, Rogers TL, Carlini AR, Brown MV 2013 Diet and phylogeny shape the gut microbiota of Antarctic seals: a comparison of wild and captive animals. Environ Microbiol 15:1132–1145. doi:10.1111/1462-2920.12022. 23145888

[10] Delsuc F, Metcalf JL, Wegener Parfrey L, Song SJ, González A, Knight R 2014 Convergence of gut microbiomes in myrmecophagous mammals. Mol Ecol 23:1301–1317. doi:10.1111/mec.12501. 24118574

[11] Kohl KD, Weiss RB, Cox J, Dale C, Dearing DM 2014 Gut microbes of mammalian herbivores facilitate intake of plant toxins. Ecol Lett 17:1238–1246. doi:10.1111/ele.12329. 25040855

[12] Moeller AH, Li Y, Ngole EM, Ahuka-Mundeke S, Lonsdorf EV, Pusey AE, Peeters M, Hahn BH, Ochman H 2014 Rapid changes in the gut microbiome during human evolution. Proc Natl Acad Sci U S A 111:16431–16435. doi:10.1073/pnas.1419136111. 25368157 PMC4246287

[13] Carrillo-Araujo M, Taş N, Alcántara-Hernández RJ, Gaona O, Schondube JE, Medellín RA, Jansson JK, Falcón LI 2015 Phyllostomid bat microbiome composition is associated to host phylogeny and feeding strategies. Front Microbiol 6:447. doi:10.3389/fmicb.2015.00447. 26042099 PMC4437186

[14] Maurice CF, Knowles SC, Ladau J, Pollard KS, Fenton A, Pedersen AB, Turnbaugh PJ 2015 Marked seasonal variation in the wild mouse gut microbiota. ISME J 9:2423–2434. doi:10.1038/ismej.2015.53. 26023870 PMC4611506

[15] Sanders JG, Beichman AC, Roman J, Scott JJ, Emerson D, McCarthy JJ, Girguis PR 2015 Baleen whales host a unique gut microbiome with similarities to both carnivores and herbivores. Nat Commun 6:8285. doi:10.1038/ncomms9285. 26393325 PMC4595633

[16] Moeller AH, Peeters M, Ndjango J-B, Li Y, Hahn BH, Ochman H 2013 Sympatric chimpanzees and gorillas harbor convergent gut microbial communities. Genome Res 23:1715–1720. doi:10.1101/gr.154773.113. 23804402 PMC3787267

[17] Moeller AH, Suzuki TA, Lin D, Lacey EA, Wasser SK, Nachman MW 2017 Dispersal limitation promotes the diversification of the mammalian gut microbiota. Proc Natl Acad Sci U S A 114:13768–13773. doi:10.1073/pnas.1700122114. 29229828 PMC5748161

[18] Moeller AH, Caro-Quintero A, Mjungu D, Georgiev AV, Lonsdorf EV, Muller MN, Pusey AE, Peeters M, Hahn BH, Ochman H 2016 Cospeciation of gut microbiota with hominids. Science 353:380–382. doi:10.1126/science.aaf3951. 27463672 PMC4995445

[19] Brooks AW, Kohl KD, Brucker RM, van Opstal EJ, Bordenstein SR 2016 Phylosymbiosis: relationships and functional effects of microbial communities across host evolutionary history. PLoS Biol 14:e2000225. doi:10.1371/journal.pbio.2000225. 27861590 PMC5115861

[20] Chung H, Pamp SJ, Hill JA, Surana NK, Edelman SM, Troy EB, Reading NC, Villablanca EJ, Wang S, Mora JR, Umesaki Y, Mathis D, Benoist C, Relman DA, Kasper DL 2012 Gut immune maturation depends on colonization with a host-specific microbiota. Cell 149:1578–1593. doi:10.1016/j.cell.2012.04.037. 22726443 PMC3442780

[21] Kumar S, Stecher G, Suleski M, Hedges SB 2017 TimeTree: a resource for timelines, timetrees, and divergence times. Mol Biol Evol 34:1812–1819. doi:10.1093/molbev/msx116. 28387841

[22] Moeller AH, Suzuki TA, Phifer-Rixey M, Nachman MW 2018 Transmission modes of the mammalian gut microbiota. Science 362:453–457. doi:10.1126/science.aat7164. 30361372

[23] Clayton JB, Vangay P, Huang H, Ward T, Hillmann BM, Al-Ghalith GA, Travis DA, Long HT, Tuan BV, Minh VV, Cabana F, Nadler T, Toddes B, Murphy T, Glander KE, Johnson TJ, Knights D 2016 Captivity humanizes the primate microbiome. Proc Natl Acad Sci U S A 113:10376–10381. doi:10.1073/pnas.1521835113. 27573830 PMC5027417

[24] Kohl KD, Skopec MM, Dearing MD 2014 Captivity results in disparate loss of gut microbial diversity in closely related hosts. Conserv Physiol 2:cou009. doi:10.1093/conphys/cou009. 27293630 PMC4806740

[25] Smith K, McCoy KD, Macpherson AJ 2007 Use of axenic animals in studying the adaptation of mammals to their commensal intestinal microbiota. Semin Immunol 19:59–69. doi:10.1016/j.smim.2006.10.002. 17118672

[26] Rawls JF, Mahowald MA, Ley RE, Gordon JI 2006 Reciprocal gut microbiota transplants from zebrafish and mice to germ-free recipients reveal host habitat selection. Cell 127:423–433. doi:10.1016/j.cell.2006.08.043. 17055441 PMC4839475

[27] Oh PL, Benson AK, Peterson DA, Patil PB, Moriyama EN, Roos S, Walter J 2010 Diversification of the gut symbiont Lactobacillus reuteri as a result of host-driven evolution. ISME J 4:377–387. doi:10.1038/ismej.2009.123. 19924154

[28] Eisen EJ 1978 Single-trait and antagonistic index selection for litter size and body weight in mice. Genetics 88:781–811. 17248819 10.1093/genetics/88.4.781 PMC1275570

[29] Bronson FH 1979 The reproductive ecology of the house mouse. Q Rev Biol 54:265–299. doi:10.1086/411295. 390600

[30] Hanshaw JB, Betts RF, Simon G, Boynton RC 1965 Acquired cytomegalovirus infection: association with hepatomegaly and abnormal liver-function tests. N Engl J Med 272:602–609. doi:10.1056/NEJM196503252721202. 14255334

[31] Chatila R, West AB 1996 Hepatomegaly and abnormal liver tests due to glycogenosis in adults with diabetes. Medicine (Baltimore) 75:327–333. doi:10.1097/00005792-199611000-00003. 8982149

[32] Abraham S, Begum S, Isenberg D 2004 Hepatic manifestations of autoimmune rheumatic diseases. Ann Rheum Dis 63:123–129. doi:10.1136/ard.2002.001826. 14722198 PMC1754901

[33] Wolpe SD, Davatelis G, Sherry B, Beutler B, Hesse DG, Nguyen HT, Moldawer LL, Nathan CF, Lowry SF, Cerami A 1988 Macrophages secrete a novel heparin-binding protein with inflammatory and neutrophil chemokinetic properties. J Exp Med 167:570–581. doi:10.1084/jem.167.2.570. 3279154 PMC2188834

[34] Tanaka Y, Adams DH, Hubscher S, Hirano H, Siebenlist U, Shaw S 1993 T-cell adhesion induced by proteoglycan-immobilized cytokine MIP-lβ. Nature 361:79–82. doi:10.1038/361079a0. 7678446

[35] Lan JG, Cruickshank SM, Singh JC, Farrar M, Lodge JP, Felsburg PJ, Carding SR 2005 Different cytokine response of primary colonic epithelial cells to commensal bacteria. World J Gastroenterol 11:3375–3384. doi:10.3748/wjg.v11.i22.3375. 15948242 PMC4315991

[36] Suzuki TA, Nachman MW 2016 Spatial heterogeneity of gut microbial composition along the gastrointestinal tract in natural populations of house mice. PLoS One 11:e0163720. doi:10.1371/journal.pone.0163720. 27669007 PMC5036816

[37] Suzuki TA, Martins FM, Nachman MW 2018 Altitudinal variation of the gut microbiota in wild house mice. Mol Ecol 28:2378–2390. doi:10.1111/mec.14905. 30346069 PMC6476712

[38] Wang J, Kalyan S, Steck N, Turner LM, Harr B, Künzel S, Vallier M, Häsler R, Franke A, Oberg HH, Ibrahim SM, Grassi GA, Kabelitz D, Baines JF 2015 Analysis of intestinal microbiota in hybrid house mice reveals evolutionary divergence in a vertebrate hologenome. Nat Commun 6:6440. doi:10.1038/ncomms7440. 25737238 PMC4366507

[39] Bindels LB, Segura Munoz RR, Gomes-Neto JC, Mutemberezi V, Martínez I, Salazar N, Cody EA, Quintero-Villegas MI, Kittana H, de los Reyes-Gavilán CG, Schmaltz RJ, Muccioli GG, Walter J, Ramer-Tait AE 2017 Resistant starch can improve insulin sensitivity independently of the gut microbiota. Microbiome 5:1–16. doi:10.1186/s40168-017-0230-5. 28166818 PMC5294823

[40] Glass EM, Wilkening J, Wilke A, Antonopoulos D, Meyer F 2010 Using the metagenomics RAST server (MG-RAST) for analyzing shotgun metagenomes. Cold Spring Harb Protoc 2010:pdb.rot5368. doi:10.1101/pdb.prot5368. 20150127

[41] Truong DT, Tett A, Pasolli E, Huttenhower C, Segata N 2017 Microbial strain-level population structure and genetic diversity from metagenomes. Genome Res 27:626–638. doi:10.1101/gr.216242.116. 28167665 PMC5378180

[42] Kumar S, Stecher G, Tamura K 2016 MEGA7: Molecular Evolutionary Genetics Analysis version 7.0 for bigger datasets. Mol Biol Evol 33:1870–1874. doi:10.1093/molbev/msw054. 27004904 PMC8210823

[43] Caporaso JG, Kuczynski J, Stombaugh J, Bittinger K, Bushman FD, Costello EK, Fierer N, Peña AG, Goodrich JK, Gordon JI, Huttley GA, Kelley ST, Knights D, Koenig JE, Ley RE, Lozupone CA, McDonald D, Muegge BD, Pirrung M, Reeder J, Sevinsky JR, Turnbaugh PJ, Walters WA, Widmann J, Yatsunenko T, Zaneveld J, Knight R 2010 QIIME allows analysis of high-throughput community sequencing data. Nat Methods 7:335. doi:10.1038/nmeth.f.303. 20383131 PMC3156573

