# Supplementary figures and images for: Experimental Evidence for Adaptation to Species-Specific Gut Microbiota in House Mice
Source: mSphere. 2019 Jul 10;4(4):e00387-19. doi: 10.1128/mSphere.00387-19 (PMC6620377; doi:10.1128/mSphere.00387-19)

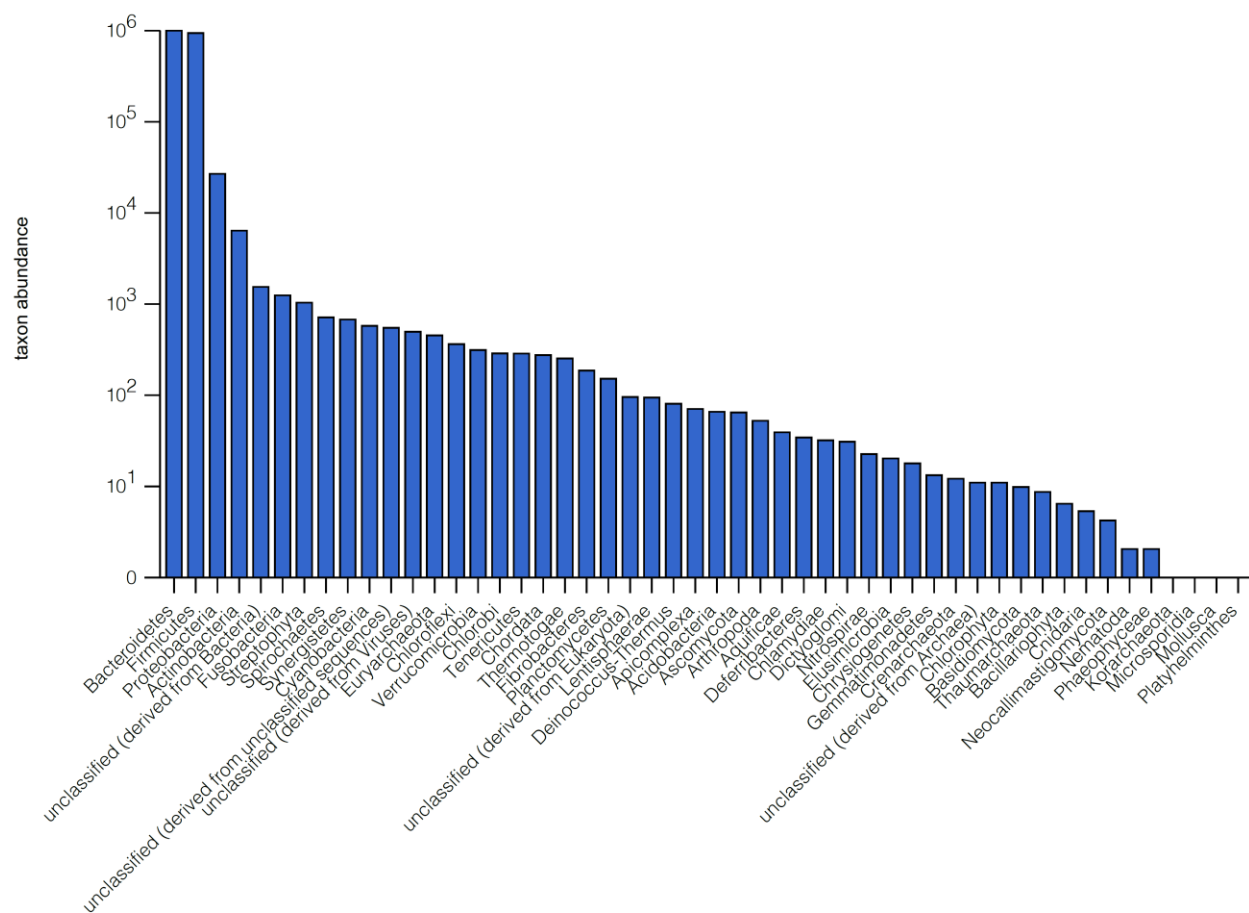

Supplement: FIG S1 [file mSphere.00387-19-sf001.pdf]

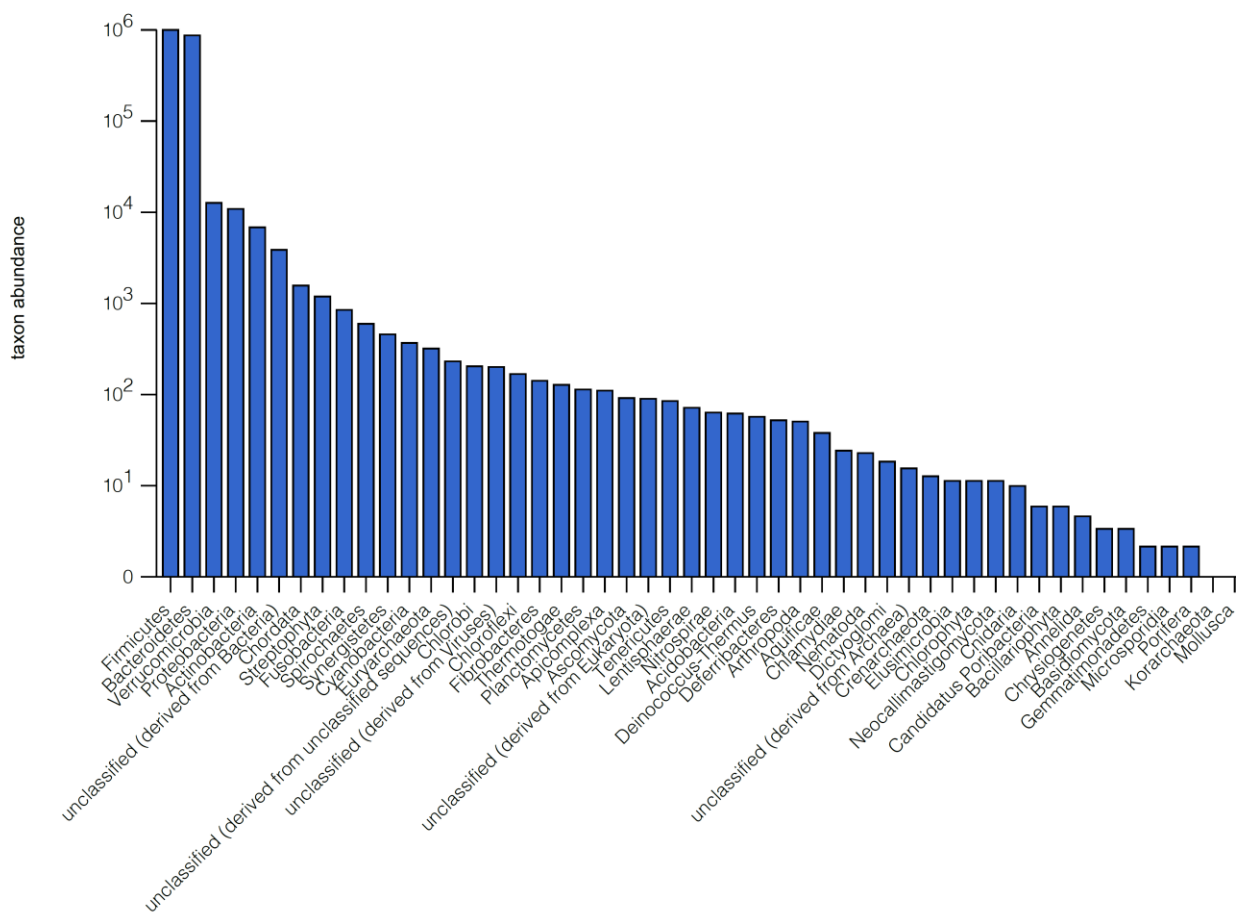

Supplement: FIG S2 [file mSphere.00387-19-sf002.pdf]

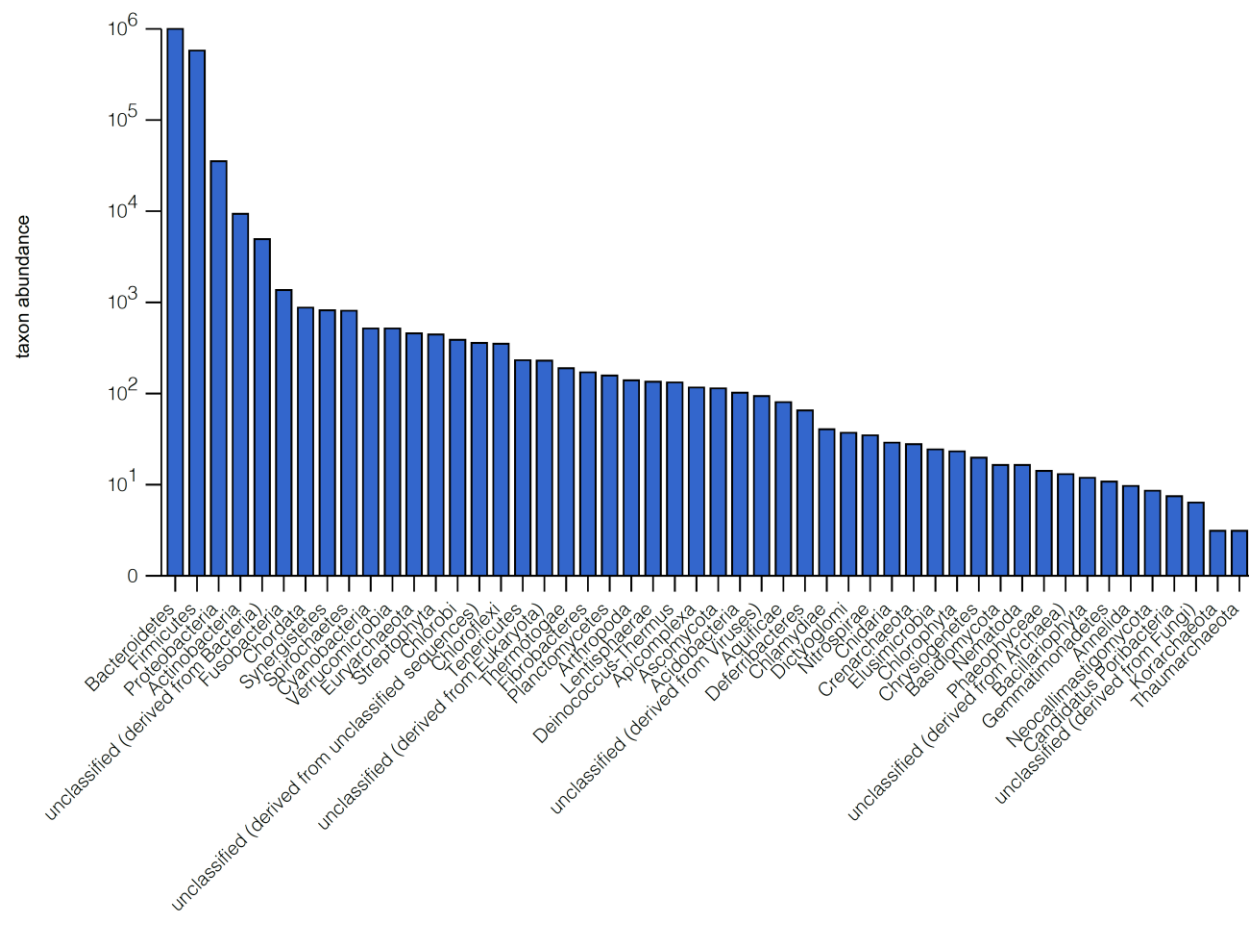

Supplement: FIG S3 [file mSphere.00387-19-sf003.pdf]

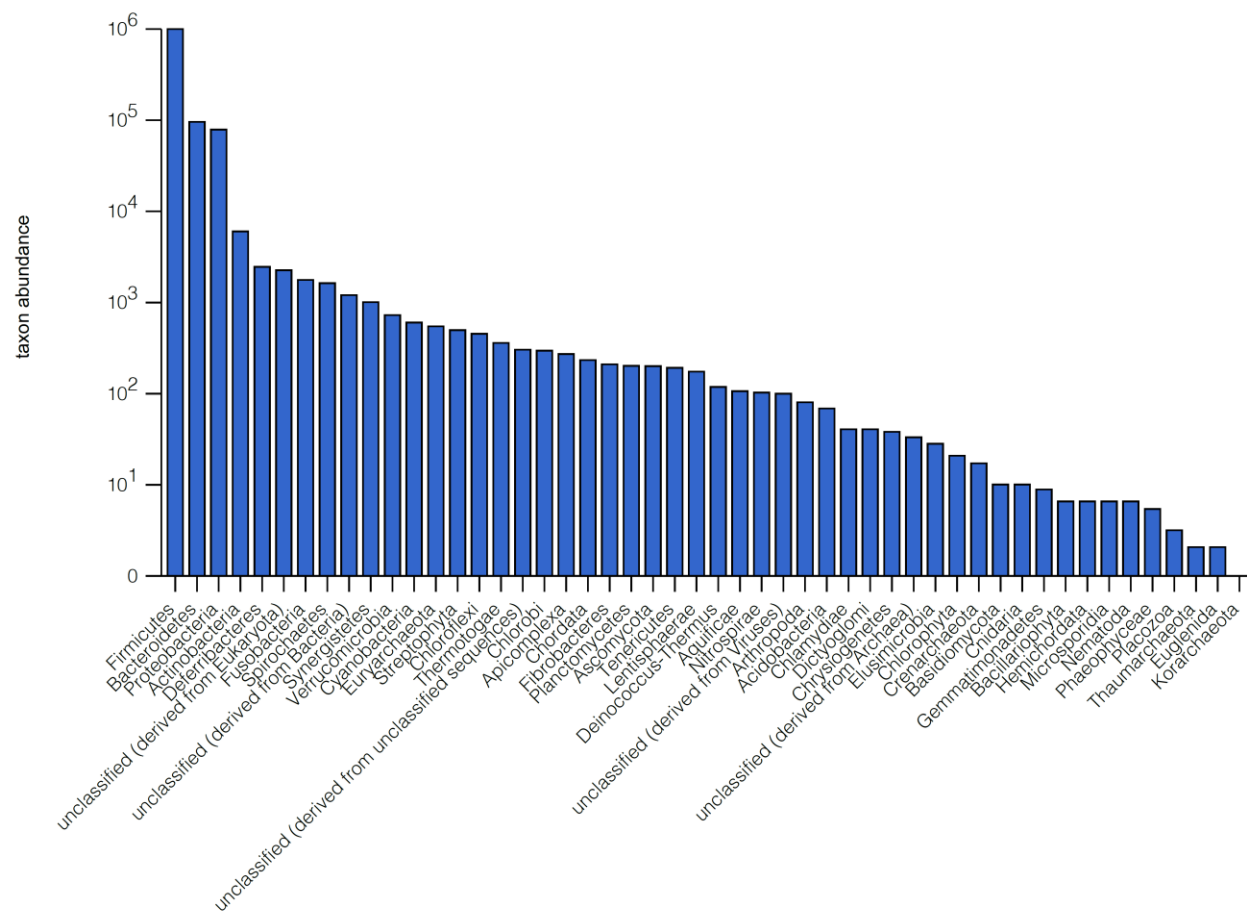

Supplement: FIG S4 [file mSphere.00387-19-sf004.pdf]

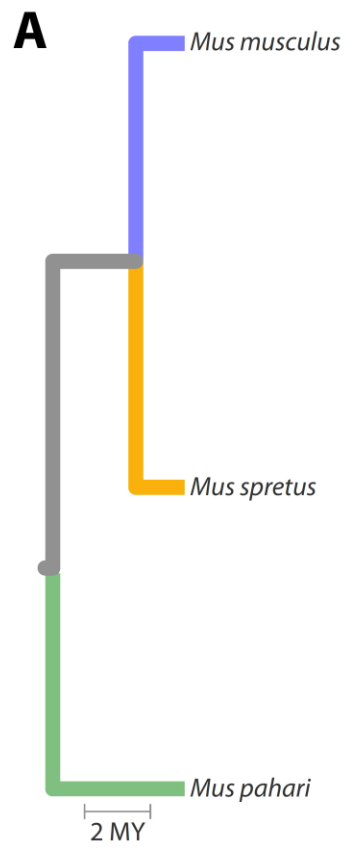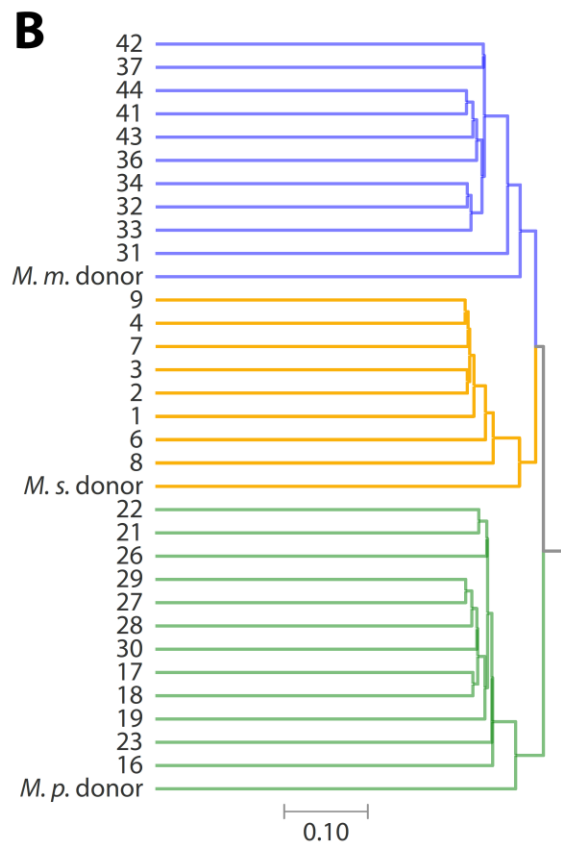

Supplement: FIG S5 [file mSphere.00387-19-sf005.pdf]

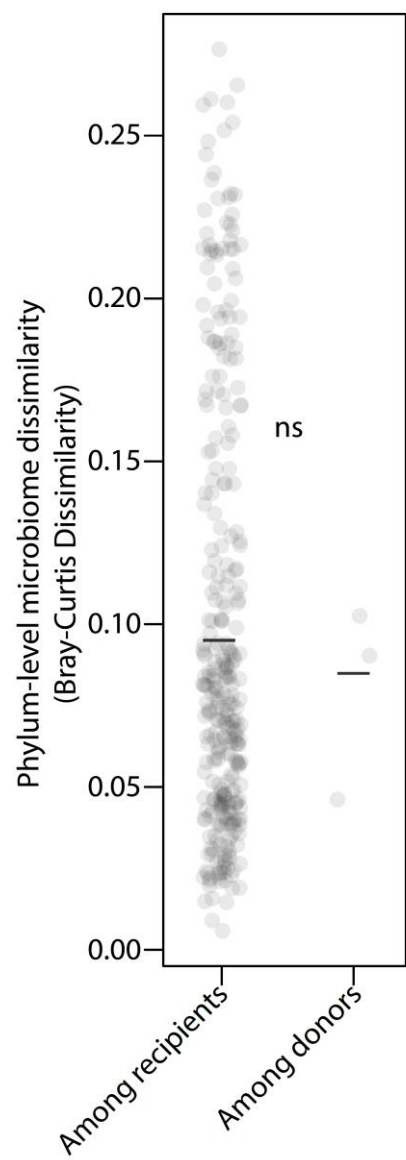

Supplement: FIG S6 [file mSphere.00387-19-sf006.pdf]
