# Supplementary material for: Experimental Evidence for Adaptation to Species-Specific Gut Microbiota in House Mice
Source: mSphere. 2019 Jul 10;4(4):e00387-19. doi: 10.1128/mSphere.00387-19 (PMC6620377; doi:10.1128/mSphere.00387-19)
Supplement: TEXT S1 [file mSphere.00387-19-s0001.pdf]

Supplementary Materials

Supplementary Methods

Food consumption.

During the experiment, per-mouse food consumption was calculated based on the weight of mouse chow provided for each cage each week minus the weight of mouse chow remaining at the end of the week. The mean chow mass consumed per mouse was 25.41g for the germ-free control, 24.89g for the *M. pahari* treatment, 25.59g for the *M. spretus* treatment, and 25.97g for the *M. domesticus* treatment. No significant differences in mean food consumption between treatments were detected, non-parametric p-value > 0.05 (t-tests for differences between means).

Statistical tests for differences in growth rate

Differences in growth rate among treatments could, in principle, be due to stochastic differences in initial body weight among treatments. For example, if all mice in the *M. domesticus* treatment were initially small (by chance), then they would exhibit "catch up growth" and display higher growth rates. Stochastic differences in initial body weight of recipients among the three microbiota treatments could lead to differences in growth rate not caused by the microbiota. To test this hypothesis, we matched individuals based on initial body weight and asked whether differences in body mass at 10 weeks of age recapitulated the pattern observed in Figure 3. Individuals displaying an initial body weight of 15-16 grams were included in this analysis (Table S2). These analyses revealed that female gnotobiotic mice that received the *M. m. domesticus* microbiota displayed increased growth rates relative to mice that received the *M. spretus* and *M. pahari* gut microbiotas (t-test p-value < 0.05). This result supports the conculsion that the microbiota treatments had differential effects on host growth rate.

In addition to analyzing a subset of individuals matched for bodyweight, we tested the statistical significance of the differences in host growth rate observed between *M. m. domesticus*, *M. spretus,* and *M. pahari* treatments through likelihood ratio tests of linear mixed effects models. To test the significance of differences in host growth rate between treatments while controlling for the potentially confounding effects of catch-up growth, we employed likelihood ratio tests of mixed-effects linear models using the lme package in R. We calculated the residuals of fold-change in body weight against initial body weight for all mice. Next, we constructed two linear mixed effects models of these residuals. The first included the donor species of the recipient individuals, the sex of the recipient individuals, and the mother of the recipient individuals as random effects. The second included only the sex of the recipient individuals and the mother of the recipient individuals as random effects. The model containing the donor species of the recipient individuals better predicted the residuals than did the simpler model (likelihood ratio test *p*-value = 0.0195). This effect of donor species was also observed when males and females were tested separately (likelihood ratio test *p*-value < 0.05 in both sexes). These results supports the conclusion that the different microbiota treatments had significantly different effects on the fold-change in body weight of germ-free recipient hosts. In addition, this result suggests that observed differences in recipient growth rate between treatments cannot be entirely explained by differences in initial body weight and subsequent catch-up growth. Repeating these likelihood ratio tests for only the recipients of the *M. m. domesticus* and *M. pahari* treatments revealed a significant effect of donor species (*p*-value = 0.0003345). However, repeating these likelihood ratio tests for only the recipients of the *M. m. domesticus* and *M. spretus* treatments revealed no significant effect of donor species (p-value > 0.05). This result supports the conclusion that *M. pahari* gut microbiota caused a significant reduction in growth

rate in recipient *M. m. domesticus* mice compared to the *M. m. domesticus* gut microbiota. However, the effect of the *M. spretus* gut microbiota on *M. m. domesticus* growth rate was not significantly different from the effect of *M. m. domesticus* gut microbiota on *M. m. domesticus* growth rate. These observations are consistent with the hypothesis that *M. m. domesticus* has adapted to its gut microbiota since diverging from *M. pahari*.
